# Prevalence and risk factors for swine influenza virus infection in the English pig population

**DOI:** 10.1371/currents.RRN1209

**Published:** 2011-02-11

**Authors:** Alexander Mastin, Pablo Alarcon, Dirk Pfeiffer, James Wood, Susanna Williamson, Ian Brown, COSI Consortium, Barbara Wieland

**Affiliations:** ^*^Veterinary Epidemiology and Public Health Group, The Royal Veterinary College, Hawkshead Lane, Hatfield, Hertfordshire, AL9 7TA, United Kingdom; ^§^University of Cambridge Department of Veterinary Medicine; ^¶^Veterinary Laboratories Agency; ^#^Veterinary Laboratories Agency - Weybridge, UK and ^**^Combating Swine Influenza Initiative consortium (under the lead of Ian H. Brown, Veterinary Laboratories Agency-Weybridge,UK, and James Wood, University of Cambridge, UK)

## Abstract

Infection of pigs with influenza viruses is a cause of considerable economic loss for pig farmers as well as a potential human health concern - as evidenced by the identification of genetic material derived from swine-adapted influenza viruses in an novel strain of H1N1 influenza virus in 2009. A study was conducted investigating the prevalence of influenza virus infection in a selection of 143 English pig herds between April 2008 and April 2009, which found evidence of recent virus circulation in over half of these herds (n=75). Farms which were sampled in the Summer months were found to have lower odds of recent virus circulation, as were farms containing pigs kept in straw yards. Additionally, farms containing pigs kept indoors and farms containing high numbers of finisher pigs per water space were found to have higher odds of recent virus circulation. It is hoped that further studies will expand on these findings, and may allow targeting of surveillance for influenza viruses in the English pig population.

## Introduction

Influenza A viruses are the cause of considerable morbidity and mortality in humans and animals worldwide. Pigs have an integral role in the ecology of these viruses, as they are susceptible to infection with both human and avian adapted strains, as well as swine adapted strains. Pigs have therefore been considered both as a hypothetical ‘mixing vessel’ for the production of new strains of virus and as an intermediary by which virus transmission between species can occur [1]. Influenza virus infection is very common in pigs, and influenza viruses have been identified as one of the most commonly isolated pathogen from outbreaks of acute swine respiratory disease [2].

Disease associated with swine influenza virus (SIV) infection of pigs has been recognised as an important cause of economic loss to pig farmers [3] [4]. There is also concern over the potential human health risks associated with porcine infection. Humans working in close contact with pigs have been reported to have increased likelihood of seropositivity to SIVs [5] [6] [7], although clinical disease and continued transmission of swine adapted strains amongst humans is rarely investigated. Despite this, pigs are proposed to have been involved in the production of the pandemic strain of H1N1 influenza virus in 2009 [8] [9] [10], with all of the progenitor viral genes identified as circulating in pigs for over ten years prior to transmission to humans [11].

Since the first identification of influenza virus infection of British pigs in 1940 [12], the recorded levels of different influenza virus strains in pigs in the country have been highly variable. Over the last 25 years, new pig-adapted strains of H1N1, H1N2 and H3N2 viruses have circulated in the British pig population [13], with some differences in epidemiology from those viruses observed in continental Europe. Of the three subtypes of influenza virus mentioned above (which comprise numerous strains), two strains (‘avian-like H1N1’ and a human-avian reassortant H1N2) are known to have been circulating in the UK pig population in recent years, and the new pandemic H1N1 2009 (pH1N109) strain has been isolated from a number of English pig herds since November 2009 [14], although the total level of exposure or infection of pigs with this strain throughout the country is currently unknown.

Since 1991, the Veterinary Laboratories Agency (VLA) has conducted passive surveillance for SIVs in the UK through virological testing of pigs with respiratory disease [13]. Although these data are useful in the identification of general trends, they cannot accurately estimate the prevalence of infection, due to the lack of denominator data and under-reporting. The most recent structured survey of SIV infection in British pigs was performed in the early 1990s [15]. Haemagglutination inhibition was used to detect antibodies to a panel of influenza virus strains in a sample of 2,000 sows randomly selected at slaughter in England and Wales. No viruses of the H1N2 subtype were included in this study as this subtype had not been identified in Britain by this time. Approximately 60% of pigs were found to be seropositive to at least one strain of influenza A virus, of which approximately 40% were seropositive to SIVs only, 15% to human influenza viruses only, and 40% to both swine and human viruses.

Despite concerns regarding transmission of SIVs to humans, few studies have investigated risk factors in pigs for infection with these viruses [16] [17] [18] [19] [20]. Farm size and the density of pigs in the area around the farm have repeatedly been identified as risk factors for influenza seropositivity of farms. It is also well recognised that the principal risk factor for initial introduction of SIV is movement of infected pigs onto a farm [21] and continued virus presence on farms has been associated with certain herd management systems [21] [22]. Risk factors for swine respiratory disease in general have been reviewed [23], and a scarcity of analytic investigations into these diseases has been identified.

Here, results are presented from a study conducted to quantify the level of recent exposure to SIVs of pigs on English farms and to investigate risk factors for farm infection.

## Materials and methods

A cross sectional study was conducted between April 2008 and April 2009, as part of a project investigating postweaning multisystemic wasting disease (PMWS) in English farrow-to-finish pig herds. Details of data collection and blood samples are described elsewhere [24] [25]. In total, 146 farms agreed to participate. Blood samples were taken from 20 pigs per farm and tested for the presence of antibodies directed against avian-like H1N1, H1N2 and human-like H3N2 strains of SIV using haemagglutination inhibition (HI) tests. The number of samples per farm for which results were available ranged from one to 24. Management data were collected through a pretested questionnaire, and data relating to the total number of pigs within a 10km radius of the farm postcode were taken from the 2004 UK agricultural census ( http://edina.ac.uk/agcensus/ ).

For the purposes of this study a reciprocal antibody titre of greater than or equal to 40 (i.e. a titre of 1/40 from serial dilution) in at least one growing or finishing pig (i.e. not from sows or weaners) was selected as indicating recent exposure of the herd to the virus. Animals for which age was not recorded (n=41) were excluded from the study, and three farms for which samples were available for fewer than five animals (n=3) were excluded from all farm-level analyses. Questionnaire and farm inspection data were entered into a relational database (Microsoft Access, Microsoft corporation), before being transferred to Stata 10.1 (Statacorp, Texas) for further data analysis. Due to the large number of variables in the dataset, knowledge of pig production systems and plausible risk factors for virus presence was used to remove variables considered highly unlikely to be associated with swine influenza status. To reduce the number of variables to be included in the formal statistical analysis stages, a three-stage process was adopted. In the first stage of this process, univariable chi-squared, Fisher’s exact tests and Mann-Whitney U tests were used to identify possible associations between variables and farm status, using a p-value of 0.25 or less to identify variables to include in the multivariable analysis.

The second stage of analysis involved grouping the resultant variables into one of ten groups relating to farm management, spatial or temporal characteristics. All variables within each group were then entered together into a multivariable logistic regression model. Ordinal and categorical variables were included as indicator variables, and a backward selection process was used to identify any associations with farm swine influenza status, using a likelihood ratio test p-value of 0.1 or less to suggest a statistical association. Ordinal variables were also tested for a linear trend in the change in the log odds of positivity across categories.

Variable groupings were then disregarded for the final stage of analysis, which first involved the placement of eight selected variables of interest (deemed *a priori* to be potential confounders or risk factors of particular interest, as detailed in Table 1) into a multivariable logistic regression model. In cases where a linear trend was considered plausible and shown to fit the data (as identified using a likelihood ratio test), the variable was entered into the final model as such. Other variables identified in the previous stage were then entered into the model using a stepwise forward selection process, starting with variables found to have the lowest likelihood ratio test p-value in the previous stage of analysis. Variables with a likelihood ratio p-value of more than 0.05, and with no evidence of a confounding effect on other associations (suggested by there being no biologically plausible confounding effect, or a change in the regression coefficient upon removal of the variable of less than 50%) were excluded from the final model. A stepwise backward selection process was then used to remove variables of *a priori* interest, according to the same criteria. Finally, an assessment was made of any plausible interaction between the remaining variables by adding interaction terms to the model and using the likelihood ratio test, with a p-value of 0.1 or less to suggest possible interaction.

### Table 1. Variables of a priori interest which were forced into the model at the start of the third stage of analysis.

**Table:** 

**Name of variable**	**Reason for inclusion in model**
Total number of pigs in the proximity of the farm*	Identified as a risk factor in previous studies [16] [17] [18] [19].
Herd size	Identified as a risk factor in previous studies [16] [18] [19]. Also considered to be a likely confounder.
Maximum number of different age groups kept on same site	Proposed risk factor in a recent study [2], and also considered to be a proxy for farm type (e.g. farrow to finish, weaner to grower etc.).
Whether herd reported moving gilts and/or boars on in the last year	Movement of animals into the herd has been identified as a risk factor in a number of reports and publications [21] [23] [26].
Accommodation types in place in herd (indoor / outdoor / straw yards)	Considered to have an effect on the transmission routes of influenza viruses between humans [27] – would be plausible to see a similar effect in pigs
Stocking density of finishers	Close contact between pigs and number of pigs per pen have been proposed as a risk factor in other reports [17] [26]. Finishers selected as another study has found that seroconversion to SIVs often occurs during the finishing period [2].

* Although this may not necessarily relate to the location of the pig sheds themselves, it was selected as a broad indicator of the density of pig production in the area in question.

## Results

In total, 2,780 sera derived from 146 farms were tested using HI (of which, 143 farms were eligible for the farm-level analysis). Using a titre of 1/40 or higher as a cut-off, Table 2 summarises the numbers and percentages of seropositive animals according to age group, aggregated across farms. It should be noted here that these do not represent the individual animal seroprevalence due to the non-random sampling procedure used within farms. As such, no account has been taken of clustering of seropositivity within farms, and confidence intervals for the age specific estimates are not given.

### Table 2. Numbers of seropositive animals amongst those sampled, according to age group

**Table:** 

**Age group**	**H1N1**	**H1N2**	**H3N2**	**At least one subtype**	**Total samples**
Weaners	14 (2%)	55 (8%)	2 (0%)	66 (9%)	711
Growers	22 (2%)	62 (7%)	0 (0%)	79 (9%)	917
Finishers	34 (4%)	67 (8%)	0 (0%)	92 (11%)	864
Sows	49 (19%)	74 (29%)	2 (1%)	97 (38%)	253

The total number of pigs present on each farm ranged from 47 to 18,000, with a median of 2,501. The number of growers or finishers tested per farm included in the study ranged from five to 23 (median = 12). The percentage of sampled growers or finishers which tested positive amongst those farms containing positive pigs ranged from 4 to 100%, as shown in Table 3.

Table 3 also shows the numbers and proportions of all 143 farms which were classified as seropositive for the three swine influenza A virus subtypes, based on results for pigs of these age groups. A total of 19 farms (13%) tested positive for both H1N1 and H1N2, and no farms were classified as seropositive for the H3N2 subtype of SIV.

### Table 3. Farm-level seroprevalence estimates for different subtypes (excluding weaners and sows)

**Table:** 

**Subtype**	Maximum within herd ‘prevalence’*	Median within herd ‘prevalence’*	**Farms positive (%) **	**95% confidence interval**
**H1N1**	0.50	0.10	30 (21%)	14 –28%
**H1N2**	1.00	0.11	64 (45%)	37 –53%
**H3N2**	0.00	0.00	0	-
**At least one subtype**	1.00	0.15	75 (52%)	44 – 61%

*This measure does not relate to the prevalence as such, but rather the proportion of sampled growers and finishers testing positive amongst those farms containing positive pigs

Univariable analysis identified a total of 39 variables potentially associated with farm swine influenza status (Table 4). The second stage, which involved multivariable analysis of these variables within each of their defined groups, identified a total of 21 variables associated with farm swine influenza status. Of these, the practice of separating boars upon entry to the farm was excluded from further analysis due to the large number of missing values, leaving a total of 18 variables, as shown in Table 4.

### Table 4. Variables associated with farm status upon univariable (all variables; p<0.25) and multivariable (variables in bold italic; p<0.10) analysis

**Table:** 

**Variable grouping**	**Variables associated in the first two stages of analysis**
Pig farm characteristics	***Total number of pigs on farm*** Number of farm sites
Sick pen management	Number of sick pens ***Sick pens in separate building to healthy pigs*** Sick pens continually occupied
Mixing and contact between pigs on farm	Use of an all in, all out (AIAO) system Pigs stay in the same building ***Number of litters mixed together*** Cross fostering ***Growers and finishers mixed together*** ***Stocking density*** (for weaners and ***for finishers***) Use of indoor accommodation for pigs ***Use of straw yards for pigs*** Use of outdoor accommodation for pigs Ventilation quality
Introduction of new pigs	Gilts separated upon entry to the farm ***Boars separated upon entry to the farm***
Feeding/water	***By-product fed to pigs*** Water location ***Pigs per feed space (for weaners and for growers)*** Pigs per water space (for growers and for finishers)*
Stress	Age of weaning Number of moves between four and 14 weeks ***Duration of rest from light*** Tail docking of piglets performed Castration of piglets performed
Contact with people	Number of new farm workers in recent years Number of other farmers visiting farm per year Number of official visitors to farm per year Use of protective clothing by visitors ***Requirement for visitors to be pig clean ***
Contact with other animals	Presence of poultry on farm Presence of cattle, sheep or horses on farm
Farmer knowledge	***Years of stockman experience with pigs*** ***Stockman participation in pig events***
Date of sampling	***Date of farm visit***

* A cut-off of 18 pigs per water space was the only category of this variable significantly associated with farm status

The final logistic regression model is shown in Table 5. This identified an increased likelihood of farm seropositivity for farms sampled in autumn, winter or spring months, for farms with more than 18 pigs per water space, and for farms rearing pigs indoors. Decreased likelihood of positivity was found for farms using straw yards (Table 5). The model fit, as identified using the Pearson chi-square test with 10 degrees of freedom, was 0.94. No evidence was found of any interaction between variables in the final model.

### Table 5. Associations with farm seropositivity, as identified in the final multivariable model

**Table:** 

**Variable**	**Odds Ratio**	**95% confidence interval**	**p-value**
***Pig access to water***			
**18 ** **finishers or less per water space**	1.00	-	-
**More than 18 finishers per water space**	5.22	1.57 – 17.43	0.01
***Season of sampling***			
**Pigs sampled in the Summer months (July-September)**	1.00	-	-
**Pigs sampled at other times of the year**	2.54	1.09 – 5.95	0.03
***Housing type***			
**No pigs kept indoors**	1.00	-	-
**At least some pigs kept indoors**	3.59	1.11 – 11.57	0.03
**No pigs kept in straw yards**	1.00	-	-
**At least some pigs kept in straw yards**	0.30	0.11 – 0.82	0.02

## Discussion

This study has, for the first time in recent years, provided insight into the seroepidemiology of swine influenza in the UK using a large sample set. Over half (52%) of the farms had evidence of ongoing virus circulation or recent virus introduction, with seropositivity in growing pigs to the H1N2 subtype most commonly identified (45% of all farms). No farms showed evidence of antibody to H3N2 in young pigs, which is consistent with passive surveillance, which has not identified this strain in the UK since 1998. There was strong evidence that farms visited in the summer months had a lower likelihood of seropositivity than those visited at other times. Regarding pig management characteristics, farms with large numbers of finishers per water space had a higher likelihood of positivity than those with fewer pigs per water space; farms containing pigs kept indoors had a higher likelihood of seropositivity than those which did not; and farms containing pigs in straw yards had a lower likelihood of seropositivity than those which did not.

A major limitation of most observational studies is selection bias. In the current study, only farrow to finish farms were included, which makes extrapolation to other farm types problematic, particularly as farrow-to-finish farms have features more likely to allow persistence of influenza virus than farms with single age groups or removal of growing pigs at weaning. It should be noted however that there was considerable variation in the management practices employed on different farms, and even between different sites on individual farms, meaning that other managemental practices were represented. As participating farms were predominantly self-selected through a vaccination scheme for PMWS, an overrepresentation of farms with a tendency towards overall poor health cannot be excluded, which may have lead to an overestimation of the true country-wide seroprevalence. Efforts were made to include high-health farms without PMWS problems (approximately 10% of farms).

An additional potential issue is that of misclassification of farm swine influenza status. The current case definition only related to infection of growers and finishers, in an attempt to identify farms with recent influenza virus circulation without resorting to more expensive virological methods. It was considered that sow and weaner serology results could not distinguish recent from historic infection, as antibodies against SIVs have been reported to persist up to 28 months in sows after removal of virus [28] and maternal derived immunity in piglets can persist up to 4 months of age [29], although an average half life of 12 days has been estimated [30]. This method would not identify farms with recent infections amongst sows (and/or weaners) only, and could result in underestimation of the true proportion of farms with virus circulation. This would be expected to be counteracted to some degree through the aggregate testing approach adopted (requiring only one seropositive animal in order to classify a herd as positive). Using the formula given in Dohoo, Martin et al. [31] (and assuming a population of infinite size), with 95% confidence, a sample of 12 pigs per farm would be expected to detect at least one infected animal assuming a prevalence of 22% or more, and a sample of five pigs per farm (the lowest number of animals tested amongst those farms included in the study) would be expected to detect at least one infected animal if the prevalence was 50% or more. Due to the generally high transmissibility of influenza viruses, it is expected that a large proportion of animals would have evidence of exposure following virus entry to a herd, provided that antibodies persist for a suitable length of time. Despite this, further investigation found weak evidence that farms testing more animals were more likely to test positive (Mann Whitney U test p=0.08).

The strains of virus used for serology in the current investigation are known to be representative of contemporary SIVs in England, thus the HI tests used are expected to detect recent infection with any of these strains. However, the possibility of infection with other, less reactive, virus strains cannot be excluded.

Antibodies to the H1N2 strain were most commonly identified in the current study, in contrast to the findings of the passive surveillance programme in the UK, which has regularly detected avian-like H1N1 virus more frequently than H1N2 until 2009 (Ian Brown, personal communication). It is difficult to compare these figures due to differences in the respective study populations. However, differences in the severity of clinical signs between the strains may result in selection bias in passive surveillance. In a field setting, variation in pathogenicity is likely to occur due to a number of factors, including virus strain. Both avian-like swine H1N1 and H1N2 have been described as being more clinically severe than classical H1N1 or human-like swine H3N2 [21] [32] [33] [34], however there are no published scientific reports directly comparing the pathogenicity of the two subtypes.

The current study is an investigation of risk factors for recent exposure of rearing pigs in pig herds to influenza viruses. This could result from either continued circulation of the virus within the herd or from recent virus introduction into the herd. Risk factors for these two events may differ, and therefore by combining both in the outcome, information regarding specific associations will be lost. However, the lack of earlier sera from the farms did not allow an evaluation of the historical swine influenza status. In the case of farms with longstanding swine influenza problems, information bias regarding farm management practices may be present, resulting from adoption of disease management processes since initial virus incursion in an attempt to control disease. No distinction was made in this study between seropositivity to H1N1 or H1N2 virus strains, as has been performed in a number of previous studies [2] [17] [19]. Although it is possible that differences exist regarding the epidemiology of the different viral subtypes, given the complex interactions between different respiratory pathogens within farms, the possible cross protective effects of different strains of influenza viruses [34] [35], and the fact that control measures for SI do not differ according to the virus strain, it was considered reasonable to assess status based on serology to both strains combined.

Manual techniques were adopted in order to reduce the number of variables prior to formal data analysis. Although this approach was based on an understanding of English pig herds and the dynamics of disease within these, it is subjective in nature, and therefore could result in bias. Other statistical techniques which could have been used for the identification of different pig herd types include scoring systems [36], multiple correspondence analysis [37], multiple factor analysis and hierarchal cluster analysis [38] [39], and multiblock redundancy analysis. These approaches were not used here as the presence of missing data for some of the variables would have led to the exclusion of a substantial number of farms.

The finding that farms visited during the summer months had a lower likelihood of positivity is consistent with the findings of others. Seasonality has been recognised as a feature of clinical swine influenza, with an increase in reported outbreaks of disease during the colder and less sunny months of the year [40] [41] and increased transmission of influenza A viruses in cold conditions [42]. Additionally, natural ventilation may be reduced in indoor herds in colder conditions, in an attempt to maintain an ambient temperature for the pigs, which may encourage virus circulation. Although it has also been shown that the virus itself can persist within herds throughout the year following introduction [43], due to the constant availability of susceptible pigs [26] [44], increased virus survival and transmission during colder months may result in a greater within-herd prevalence, and therefore a greater likelihood of detection during this time.

Pig access to water was strongly associated with swine influenza status, with farms with large numbers of finisher pigs in relation to water sources having a higher likelihood of positivity. This finding may relate to both pig-pig contact and resultant social stress factors. A large number of pigs per water source would be expected to increase both direct and indirect contact between pigs, which may aid transmission of influenza viruses. Reduced access to water may also increase stress amongst the animals, which has recognised immunosuppressive effects and may therefore also encourage persistence of virus within the herd. The finding of this association amongst finishers in particular may be a result of the criteria used for classifying a farm as positive for virus circulation. The possibility of this association being a result of confounding by other variables, cannot be excluded. However, plausible confounding variables for this association such as stocking density were included in the final model and not found to be associated with herd status, which points away from a confounding effect.

The finding that farms rearing pigs in indoor pen-based systems have an increased likelihood of positivity may relate to higher stocking densities and lower levels of ventilation, which are recognised risk factors for transmission of respiratory pathogens between pigs [23] [45], although a variety of pen-based systems were included in the definition here (combining those with and without individual kennels). Confined animal feeding operations (‘factory farms’) have been proposed to be involved in the amplification of influenza viruses in a recent report using mathematical modelling techniques [46], although no comparison was made in this report with other farming systems. Increased stocking density was found to be strongly associated with keeping pigs indoors (chi-square p<0.001) in the current study, with 100% (n=22) of herds with a ‘high’ stocking density amongst finisher pig containing animals kept indoors (27% of these herds also contained pigs in straw yards and 9% contained pigs kept outdoors). Although stocking density itself was not found to be associated with the circulation of influenza viruses in the final model, a study in Belgium has found that the number of pigs per pen was positively associated with swine influenza H3N2 seropositivity [17]. Finally, it should also be noted that there was a strong association between the use of indoor pen-based systems and outdoor systems, with only 14% (n=16) of farms rearing pigs indoors also keeping pigs outdoors (chi-square p<0.001). As such, it is plausible that there is collinearity between these variables, and the possibility of an additional protective effect of keeping pigs outdoors cannot be excluded.

The protective effect of keeping pigs in straw yards has not been found in other studies. Further investigation of this variable found that only 24% (n=10) of these farms used straw yards for all three age groups, which demonstrates the variability in management practices amongst those farms classified as ‘straw yards’ (conversely, 62% (n=68) of farms keeping pigs indoors kept all three age groups indoors). This ‘mixing’ of exposure makes it difficult to attribute a direct effect of straw yards. Pigs kept in straw yards would be expected to receive some degree of shelter from inclement climatic conditions, whilst still being kept at relatively low stocking densities. This finding warrants further investigation.

Introduction of virus onto a farm is most commonly associated with the introduction of an infected animal [19] [21] [23] [26]. However, no evidence was found of an association between recent movement of animals into the herd and farm status. As well as limitations in the study regarding misclassification, as described above, this lack of association may be due to many farms experiencing persistent infection rather than recent introduction of infection through infected animals, thus masking a potential association. This would also explain the lack of observed association with the frequency of movement of non-farm workers onto farms and into pig buildings, which was postulated to be a risk factor for virus entry *a priori*, and has been observed to be a risk factor for influenza virus presence in Malaysian pig farms [19].

No association was observed between herd size and/or pig density in the region and farm status in the final model, which were identified as risk factors for infection in a number of other studies [16] [17] [18] [19]. Plausible reasons for an association between herd size and pig disease have been detailed in a recent review [47], and include biological, managemental and diagnostic mechanisms, whereas little is currently known regarding the spatial pattern of influenza virus spread between herds in a geographical area.

## Conclusions

This study is one of the first in recent years to estimate the seroprevalence of SIVs amongst English pig herds, and is the first to identify risk factors for the presence of SIVs in these herds. Further studies are required to confirm the findings. The potential human health risks associated with SIVs are well recognised [11], as evidenced by the recent emergence of pandemic H1N1 (2009) virus in humans, which was found to contain a genetic component from swine adapted viruses. Although serological data is useful for the investigation of SIVs in circulation in pig populations, virological surveillance is required in order to monitor virus evolution in this species. The passive virological surveillance system for SIVs currently in place in the UK is likely to be subject to considerable selection bias as it depends on sampling diseased pigs. Consideration should therefore be given to the implementation of structured serological surveys to complement this surveillance stream, possibly targeting surveillance to high-risk farms based on risk factors identified using analytic studies such as the current one. In order to facilitate these investigations, further work is required in order to accurately classify English pig herds with respect to farm practices.

## Acknowledgements

The authors would like to thank BPEX for the support given to this project through their vaccination programme, as well as the vets who assisted with the collection of blood samples. Special thanks go out to Martina Velasova, who collected a large amount of the data and many of the blood samples, and Alexander Holland of the VLA, who organised the testing of the blood samples. Finally, we would like to thank all the farmers who agreed to participate in this study. 

## Funding information

Initial data collection for this project was funded by a grant (BB/FO18394/1) from the BBSRC CEDFAS initiative, BPEX Ltd, BioBest Laboratories Ltd. and Pfizer Animal Health Ltd. Additional funding for further blood testing and analysis came from a grant from the BBSRC (BB/H014306/1). JLNW is supported by the Alborada Trust and the RAPIDD programme of the Science and Technology Directorate, Department of Homeland Security.

## Competing interests

The authors have declared that no competing interests exist.
